# Health literacy and related behaviour among pregnant women with obesity: a qualitative interpretive description study

**DOI:** 10.1186/s12884-022-05023-0

**Published:** 2022-09-19

**Authors:** Maiken Meldgaard, Annesofie Lunde Jensen, Amalie Damgaard Johansen, Rikke Damkjær Maimburg, Helle Terkildsen Maindal

**Affiliations:** 1 Department of Public Health, Aarhus University, Aarhus, Denmark; 2 Steno Diabetes Center Aarhus, Aarhus, Denmark; 3 Department of Clinical Medicine & Department of Obstetrics and Gynaecology, Aarhus University Hospital, Aarhus, Denmark; 4 School of Nursing and Midwifery, Western Sydney University, Sydney, Australia

**Keywords:** Health literacy, Pregnancy, Obesity, Overweight, Prevention, Health care service

## Abstract

**Background:**

Obesity in pregnant women is increasing worldwide, affecting the health of both mother and baby. Obesity may be associated with inadequate health literacy, a central competence when navigating antenatal health information and services. This study explores women’s health literacy by examining their knowledge, motivation and skills to access, understand and evaluate health information and the related behaviour among a sample of pregnant women with a prepregnant body mass index (BMI) > 25 kg/m^2^.

**Methods:**

An inductive, qualitative study using an interpretive description methodology. Data was collected through ten semi-structured interviews with pregnant women with a prepregnancy BMI > 25 kg/m^2^ attending antenatal care at the midwifery clinic at Aarhus University Hospital in the Central Denmark Region.

**Results:**

Pregnant women with obesity understand general health information provided by health professionals, but translating this knowledge into specific healthy behaviours presents a challenge. Although difficulties navigating booking systems and available digital services contribute to this problem, apps can help facilitate navigation. However, successful navigation may depend on adequate e-health literacy. Conflicting information from health professionals, social media and families also present a challenge for pregnant women, requiring a broad skillset for critical evaluation and resolution.

**Conclusions:**

Adequate health literacy is necessary for pregnant women receiving antenatal care to (i) translate general health information into personalised healthy behaviour, (ii) access and navigate complex and digitalised systems, and (iii) critically evaluate conflicting information. Person-centred differentiation in the organisation of antenatal care may benefit vulnerable pregnant women with inadequate health literacy.

**Trial registration:**

The study was registered cf. General Data Protection Regulation, Aarhus University Journal number 2016–051-000001, serial number 1934.

**Supplementary Information:**

The online version contains supplementary material available at 10.1186/s12884-022-05023-0.

## Introduction

Social inequality in health continues to increase among pregnant women worldwide, including access to and use of maternity care services [1–3]. Due to the increasing emphasis on an individual’s role and responsibilities in their health care, health literacy has gained importance on the European and global health agenda [4–6]. Health literacy is defined as the ability to access, understand, evaluate and communicate health information to promote, maintain and improve health in various settings across the life course [7]. As a multidimensional, changeable concept encompassing the interacting capacities of pregnant women, health professionals and services, health literacy has been recognised as a critical factor in reducing health inequality [8]. A high degree of health literacy and self-care is required to successfully navigate, understand and convert health information during pregnancy [9–11].

Given the increasing prevalence of obesity among pregnant women, pregnant women with a body mass index (BMI) above 25 kg/m^2^ are a high-risk group warranting particular attention. In the United States, 39% of pregnant women aged 20–39 years were classified as obese in 2017–2018 [12]. In Denmark, the percentage of overweight or obese women of childbearing age has increased from 39% in 2010 to 44% in 2017 [13]. Overall, almost 50% of women in developed countries begin their pregnancy with a body mass index (BMI) above 25 kg/m^2^ [14, 15]. Women with obesity have a higher risk of complications during pregnancy and childbirth than women with a normal BMI [16], and obesity in pregnancy is associated with unfavourable health outcomes for both mother and child. Maternal complications include an increased risk of gestational diabetes mellitus (GDM), preeclampsia, metabolic syndrome and later-life stroke [17, 18], and children born by women with obesity have an increased risk of obstetric morbidity, childhood and adulthood obesity and later-life development of diabetes mellitus [19, 20].

Low health literacy in pregnant women – defined as an inability to find, understand, navigate and apply health information during pregnancy – may be associated with maternal obesity [21]. Both low health literacy and obesity can cause complications during pregnancy and birth for both mother and child. Increased health literacy has been suggested as a way to reduce the long-term consequences of maternal obesity [22, 23]. Pregnancy may represent a “teachable moment” for health promotion interventions, since nulliparous women are preparing to adopt the maternal role for the first time and may have concomitant expectations of lifestyle and self-image changes [24].

Research on health literacy among pregnant women with obesity remains limited, however [25]. A more comprehensive understanding of pregnant women’s health literacy experiences is crucial for identifying their needs and skills in relation to antenatal care. Increased knowledge of pregnant women’s health literacy experiences may help reduce inequality in pregnancy and guide healthcare systems’ maternity-care plans [26]. Exploring pregnant women’s experiences of health literacy may help identify health literacy barriers and struggles from the women’s point of view, which can be valuable knowledge to the clinical practice.

Our study’s objective is to explore (i) the knowledge, motivation and skills of pregnant women with obesity to access, understand and evaluate health information during pregnancy, and (ii) how their knowledge, motivation and skills impact their health behaviour in connection with health professionals, services and information.

## Methods

### Study design

We designed this study as an inductive, qualitative study using an interpretive description (ID) methodology. Developed by Thorne, the ID methodology was chosen for its explorative nature, allowing researchers to generate knowledge relevant to clinical practice [27, 28]. By examining health literacy from the perspective of pregnant women with obesity, we aimed to generate knowledge supporting a person-centred preventative maternity service. ID is particularly suitable for generating knowledge relevant to the clinical context and practice of applied health disciplines, as it can address complex experiential questions such as pregnant women’s health literacy experiences in relation to maternal services and care. Furthermore, ID allows us to examine the topic through a theoretical lens. In this study, we applied theories of health literacy for developing the interview guide [29].

### Setting and informants

We undertook the study in the Central Denmark Region, one of five administrative regions in Denmark. Due to the COVID-19 pandemic, interviews were conducted via online media such as Zoom, Skype or Facetime.

We used purposeful sampling to select nulliparouspregnant women with obesity for study participation [27, 30]. The study sample was selected to investigate the health literacy behaviours of pregnant women with a pre-pregnant BMI > 25 kg/m^2^. Danish-speaking nulliparous women with a pre-pregnancy BMI of *>* 25 kg/m^2^, a minimum age of 18 and at least 20 weeks of gestation were eligible for participation. A priori, we decided to conduct between 10 and 20 interviews, based on available resources and the duration of the study. The participants included showed to be good informant as they were well articulated and make thorough decribtion during the interviews. This leed to strong and clear communication between researchers and participants and an achievement of high informational power [31]. Hence, after the first ten interviews evaluation found a valid interpretation of the phemonen studied [27].

Due to the COVID pandemic, participants were recruited from the Aarhus University Hospital (AUH) Antenatal Midwifery Clinic’s Facebook page. Women willing to participate in the study were invited to contact the research team, who forwarded the relevant project information, including a study description and consent form. A.D.J. or M.M. then telephoned potential participants to schedule convenient interview dates with them. Interviews were only initiated once participants had signed an informed consent declaration and the interviewer was confident they understood the nature and scope of study participation. Current antenatal practice and frequently used terms are described in Table 1.

**Table 1:** Description of current antenatal practice and clarification of terms

Term	Description and clarification
Antenatal care and services	Pregnant women in Central Denmark Region are enrolled in a basic antenatal course, including five visits to The Antenatal Midwifery Clinic (week 18, 28, 35, 38 and 40). Women expecting their first child are invited to participate in a birth and family preparation course.
Emento	A digital platform developed by Emento® for information and communication between health professionals and the pregnant women attending antenatal care in The Antenatal Midwifery Clinic. Pregnant women have access to the platform through an app. Approximately 85% of pregnant women use the Emento app during pregnancy.
Health Professionals	Health professionals whom pregnant women interact with during pregnancy and birth, including general practitioners, midwives, obstetricians, nurses, and health nurses.
Booking systems	General practitioners refer the pregnant women to The Antenatal Midwifery Clinic, which sends out invitations for ultrasound scans and midwifery appointments. All pregnant women receive notification of the time and location of their antenatal appointments. Women can see or change booked appointments on a webpage.

^a^Information available on the webpage of Aarhus University Hospital www.auh.dk2021 [Available from: https://www.auh.dk/afdelinger/kvindesygdomme-og-fodsler/graviditet-og-fodsler/]

### Data collection

Interviews were conducted between April and December 2020 by A.D.J. or M.M.

The research team developed a semi-structured interview guide consisting of topical research questions and underlying interview questions, which were pilot tested among peers before use. Questions were generated based on scales included in the Health Literacy Questionnaire (HLQ) [29] and existing literature. Two example questions from the interview guide include:

(i) Do you consider which health professionals to contact if you need someone to talk to about your pregnancy? If yes, what are your thoughts about this? How do you reach them? Are you clear about when a midwife is best placed to help you and when a doctor is more appropriate?
(ii) What is your experience of navigating the health care system, both practically and in relation to the information advice and recommendations you receive? Do you find it hard or easy? How do you navigate and orientate yourself?

For elaboration, see Supplementary Table 1.

The majority of questions were deliberately kept open to minimise bias and allow participants the space and flexibility to answer freely and fully. We used open-ended interview questions to explore and describe the pregnant womens’ views, experiences, and perceptions of their health literacy behaviour. We also collected participants’ self-reported demographic data before interview, including height and weight (for calculating BMI).

All interviews were recorded with a dictaphone. The interviewer followed the interview guide and was responsible for time management. However, questions were allowed to develop naturally based on the information provided by the respondent. At the end of each interview, the researcher outlined the most significant elements of each interview and wrote up a brief reflective summary of the overall interview experience, any key discussion topics that arose, and the respondent’s emotional responses and reactions during the interview. Interviews were digitally audio-recorded with consent and transcribed verbatim in timespans in NVivo. Interviews lasted for approximately 1 h.

### Analysis

A.D.J. and M.M. transcribed and coded the empirical data in NVivo. The first step in the analysis involved repeated reading of all transcribed interviews. M.M. and A.L.J. undertook this process independently of each other. Data collection and analysis occurred concurrently, as per the ID methodology, and the material was coded bottom-up via direct generation of codes from the data. Once the two research members completed the initial coding, they collaborated to compare coding names and consistency.

After the initial coding process, we began cataloguing codes to identify patterns or themes in the data. A constant comparative analysis was undertaken whereby researchers could redefine or restructure themes at any point during the analytic process.

## Results

A total of ten pregnant women with a pre-pregnancy BMI ≥ 25 kg/m^2^ and aged between 23 and 41 years participated in this study. All were nulliparous women in gestation week 20 or more. Participant characteristics are presented in Table 2.

**Table 2:** Participant characteristics

Identifier	Ethnicity	Age	Employment/education
ID1	Turkish	33	Medical laboratory technologist
ID2	Danish	23	Nurse student
ID3	Danish	41	Doctor
ID4	Danish	30	Kindergarten teacher
ID5	Danish	28	Media and communications student
ID6	Danish	26	Social worker
ID7	Danish	28	Unemployed
ID8	Danish	33	Teacher
ID9	Romanian	34	Health professional
ID10	Danish	24	Nurse student

We identified three main themes from the interviews: (i) a need for clear and specific action-oriented knowledge, (ii) difficulties navigating a complex health system, and (iii) difficulties reconciling conflicting information and options.

### The need for clear and specific action-oriented knowledge

Pregnant women are motivated to search and consult multiple sources of information. They understand the general health information provided by health professionals and feel well-informed about general health recommendations.

“*I am a bit of a tech nerd. I frequently use apps, the internet and social media. For example, I use Emento a lot.”* ID5

“*I think they’ve done a good job informing me. Initially, they said do you know Emento (app)? I think the amount of information I received has been sufficient. Not overwhelming.* “ ID1

Although participants reported receiving sufficient advice from health professionals, feeling well informed, and utilising multiple sources of information, they struggled to translate general health information into healthy everyday-life behaviours.

*“I missed receiving a few proper recommendations about things. I don’t know if I should just rely on my commonsense or what. I didn’t receive more specific information. I had to search all kinds of blogs, web pages, etc. But it could be something directly from the health authorities or the health professionals, e.g. the physician or midwife.”* ID7

*“I think that recommendations have been very general – including recommendations for daily exercise. You then have to find out which kind of exercise you need, and that’s not very specific.”* ID6

Hence, participants’ knowledge was not necessarily reflected in their behaviour, and they expressed a need for more specific action-oriented knowledge. For example, ID2 explains that, despite knowing that physical activity is recommended during pregnancy, her husband’s daily use of their only car limits her options for accessing physical activity classes, courses or locations. We also identified a need for specific information about which types of physical activity are suitable during pregnancy and how best to undertake them.

*“When I cannot go for a walk or bike ride due to back pain, what should I do then?* “ID4

*“I feel left alone with this part. I have to find out on my own what works for me. Especially during pregnancy. What exercise am I supposed to do? “*ID8

The receipt of test results is another example of the gap between the information provided by health professionals and pregnant women’s ability to receive, understand and apply it. Participants receive information from health professionals about their BMI status and glucose tolerance but are unsure how to understand and respond to these details. Aware that these results might negatively influence both their own and their child’s health but unsure how to positively change their behaviour, they require more support and guidance from health professionals.

*“When they told me that my BMI was already too high at the beginning of my pregnancy, I was surprised they didn’t offer any recommendations for improving it. Should I be more careful about what I eat? I know that some women gain a lot of weight [...] – should I have eaten in a different way? Should I have been more careful? Should I have exercised more?”* ID9

“*Mostly related to my result on the glucose tolerance test. They didn’t clarify its meaning. They didn’t explain the result, but wrote that I should be attentive to diet and physical activity. I thought that it would be nice to receive some explanation. Is it because I’m at risk of diabetes during my pregnancy? It would be nice to receive some more information.”* ID8

ID8 expresses a need for further explanation with a personalised “how-to” focus, illustrating a desire for a more precise explanation of what such results mean for pregnancy and everyday life and whether or not there is a need to change behaviour accordingly.

In summary, there is a gap between pregnant women’s knowledge and their ability to change their behaviour accordingly, despite their motivation.

### Difficulties navigating a complex health system

Participants described positive experiences using the Emento app, which guides and supports pregnant women in navigating health services during pregnancy. However, participants also described difficulties navigating booking systems and understanding when and how to book particular types of appointments, which they experienced as barriers to fully utilising antenatal services.

*“I think that it could be easier to navigate the booking system, or perhaps we could be given better instructions for using it. “*ID4

*“It’s been unclear sometimes whether I should book appointments myself or wait to be invited. An example is my glucose tolerance test; I had to figure out whether it would happen at my doctor’s surgery or the hospital. I asked if I should book the appointment myself, as I hadn’t received any information about it.”* ID9

“*A guide or timeline that explained what I was supposed to do in which week would have been beneficial for me. I’m sure that such a guide exists. I just didn’t receive one.*” ID10

These examples suggest that the organisation of antenatal appointments is not always adequately managed by the clinic or other health professionals. Such ambiguity presents a challenge for pregnant women, who feel responsible for booking appointments and utilising available services at the right time via a navigation process that is not transparent for them.

In summary, participants described difficulties successfully navigating antenatal booking systems and services. Therefore, not all pregnant women can meet navigation requirements, leading to uncertainty and disorientation. Participants expressed a need for health professionals to accommodate their navigation barriers and help them manage their pregnancy course.

### Difficulties reconciling conflicting information and options

There is a wealth of conflicting advice about how to behave “correctly” during pregnancy. Participants reported receiving numerous different opinions from their social network, family, health professionals and via social media.

*“There are many different opinions about how you should behave when pregnant. You should do this and that. “*ID6

Pregnant women’s ability to critically navigate conflicting advice and recommendations is therefore essential. However, even pregnant women with a health professional background might find such conflicting information challenging.

*“Even as a health professional myself, I find it hard to navigate.”* ID3

Pregnant women encounter many different health professionals during their pregnancy. Participants described challenges in knowing whom to seek clarification from about conflicting information.

*“They say that you should just ask your midwife, and I think to myself, ‘who is that?’”* ID5

Uncertainty about whom to ask for advice about conflicting information presents a challenge for pregnant women. The above example suggests that ID5 encountered numerous midwives during her pregnancy but did not feel she had a relationship with any particular midwife. Confusion about which midwife to ask may impact a woman’s ability to interact with health professionals when necessary.

Some participants’ experiences suggest that the various antenatal health professionals they interact with are not adequately collaborating and communicating with one another.

*“I explained to my physician about the test my midwife performed, but I don’t think she had any understanding of it. It was as if she didn’t know about the test. “*ID2

The Covid vaccination is an example of a topic that participants felt particularly conflicted over. Even though their health professionals recommended the vaccination, they reported strong and conflicting opinions from their family and social network.

*“I have many family members who are against vaccination. I cannot decide what is right or wrong.”* ID6

*“Then a family member sends you 10,000 posts and pictures about the side-effects of vaccination.”* ID1

These accounts suggest that family members’ opinions may significantly influence pregnant women; they are unsure whether to trust information received from health professionals if it conflicts with the opinion of one or more family members.

However, others report that they reconcile such contradictions by trusting information received from their health professionals. Hence, not all participants experienced the same level of struggle in response to conflicting advice. This difference may suggest different levels of health literacy among pregnant women.

*“I think you should distinguish between the information you receive from a health professional and, for example, the information you receive from your friend about her opinion on something.”* ID3

*“When it is from my social network, I am friendly and say that it was nice of them to share, but I don’t immediately believe it as others might do. I consider it critically before I make any decisions.”* ID2

Contradictions between the advice offered by health professionals versus that offered by family, friends or social media present a clear challenge for pregnant women.

## Discussion

### Implications for practice, policy and future research

In the following we compare our results to existing evidence of health literacy among pregnant women. However, when interpreteting the results presented in this study, the context needs consideration. Health literacy is a multidimensional, changeable and context dependent concept. Thus, the results in this study are marred by the context within the Danish healthcare system, The Antenatal Midwifery Clinic and organization of antenatal care. On the other hand, as shown below, the results from this study holds similarities with studies from other countries, indicating that findings have representativity within a global context. Thus, findings may be generalizable to other antenatal care practices with similar setting and organization.

### A need for clear and specific action-oriented knowledge

We found that participants feel well-informed about general health recommendations in pregnancy. The pregnant women in this study were interested and motivated, consulting multiples sources of information. This finding is consistent with a study by Garnweidner et al., who found that pregnant women actively sought information about nutrition [32]. Vamos et al. also found that pregnant women described rich, contextual health literacy experiences [25].

However, multiple studies have found that pregnant women struggle to understand health information [33–36]. In our study, participants struggled to align their behaviour with their health knowledge, suggesting that adequate knowledge about health does not necessarily impact the women’s behaviour. The digital apps may be effective in providing the women with general health information but the women needs time with and support from their health professionals to obtain individual health information to change behaviour in alignment with recommendations and their knowledge. Wilhelmova et al. reported similar results: although the pregnant women in their study received adequate health information, most of their lifestyles remained far from optimal [37]. Compared to women with adequate health literacy, Poorman et al. also found that pregnant women with limited health literacy were less likely to take a daily vitamin pill during pregnancy or breastfeed their child [38]. Moreover, a Danish study found that, among pregnant women with GDM, those with limited health literacy struggled to implement the recommended behaviour changes [39].

Pregnant women in this study experienced recommendations about health-related topics such as diet and physical activity as general and non-specific. Similar results were reported by Garnweidner et al. [32], whose study of first-time pregnant women with a pre-pregnancy BMI above 25 kg/m^2^ showed that participants perceived health information as very general and focused on food safety.

Although the women in our study were informed about their BMI and glucose tolerance results, they lacked confidence about how to act on them, requiring specific behaviour-oriented knowledge and advice. Pregnant women with limited health literacy need individual guided support to archive positive behaviour change. Sahin et al., found that pregnant women with high health literacy led more health-promoting lifestyles [40]. It is essential that health professionals support and guide pregnant women with information, goal setting, planning and follow-up to help them respond to unfavourable health choices and behaviour. There is a need for health promotion and care to support healthy behaviour development.

### Difficulties navigating a complex health system

Some of the women in our study struggle to navigate the booking systems. They feel that it is their responsibility to book appointments and, therefore, their responsibility when they miss or fail to book them.

As booking systems and reminders are often digitalised, pregnant women need specific skills to interact with the associated technology. Women with limited e-health literacy (the ability to seek, find, understand, and appraise health information from electronic sources and apply the knowledge gained to addressing or solving a health problem [41]) may find this challenging. This offers a possible explanation for the barriers some women experience when booking appointments during pregnancy. For example, Brusniak et al. found that most pregnant women in their study showed low compliance and engagement with digital monitoring [42]. Health professionals are aware of this problem. A systematic review published in 2020 concluded that midwives are ambivalent about using e-health technologies as a one-size-fits-all communication platform in antenatal care and point out the risk associated with it [43]. There is thus a need for further research into e-health literacy and the barriers pregnant women experience with booking antenatal appointments as variables affecting their ability to navigate a complex health system. Health professionals must be educated to identify and support different levels of e-health literacy among pregnant women. Hereby increasing health professional’s foundation to support and guide pregnant women, who struggle with use of e-health and navigation.

### Difficulties reconciling conflicting information and options

Our study suggests that women receive conflicting information during pregnancy and may struggle to critically evaluate and reconcile these conflicts. Similar results were found in other studies of pregnancy-related health topics, including medication use, diet and physical activity [44–47]. For example, Findley et al. found that decision-making about physical activity during pregnancy was influenced by pressure from others, pressure from social media and beliefs about physical activity [46].

Participants mentioned conflicting advice about the Covid vaccination as a particular challenge in this respect. Similarly, several studies have shown that pregnant women experience a wealth of contradictory opinions and guidance about other vaccination programmes during pregnancy [48–50]. This inconsistency may be problematic if women choose to trust information from their social network over information from health professions, with potentially consequences for both the mother and child’s health. Knowledge of distributed health literacy (defined as health literacy distributed through family, friends and network) among pregnant women may help understand how they reconcile conflicting information. Distributed health literacy may also provide a valuable resource for understanding decision-making about health [51].

Pregnant women engage with many different midwives at The Antenatal Midwifery Clinic in Aarhus during their course of pregnancy. A review by Sandall et al. suggests that midwife-led care and continuity increases satisfaction with care, information, and decision-making [52]. The midwifery-led organisation of antenatal care may also help address problems arising from the conflicting information and opinions women are subject to during pregnancy. In addition, health professionals must recognise and accommodate women’s differing critical appraisal capacities for responding to conflicting information. Health professionals need to address these challenges and provide the information pregnant women need to make informed choices. However, they should also be aware that such challenges affect women in different ways. Some of our participants described using what they called a “critical view” and trusting health professionals’ recommendations. Different levels of health literacy among pregnant women potentially explain their varying skills in responding to contradictory information and opinions during pregnancy [5]. The Danish Health Authority suggests reorganising antenatal care according to four socioeconomic-and-obstetric-risk levels to support women’s different antenatal needs [53]. Differentiation would help support all levels of health literacy, potentially ameliorating the rising social inequality in health [54].

### Strength and limitations

Our study design provided unique insight into women’s experiences of their health literacy behaviour during pregnancy. The application of Interpretive Description methodology provided a conceptual description and in-depth interpretation of relations and patterns within the practice phenomenon of the health literacy concept. Hereby, new insights, comprehension and knowledge specific for the practice of antenatal care emerged. Thus, results can be brought back and help improve and qualify practice.

Interviews were necessarily conducted online because of the global Covid pandemic, which may have affected their content. Physical separation of the interviewer from the interviewee introduced a risk that they missed important non-verbal cues and information, e.g. body language. Also, there are potential limits to establishing trust and connection between interviewer and interviewee in a virtual setting [55]. However, conducting carefully planned interviews under such circumstances can also be considered a strength, as it may have encouraged a more open-minded response to the interview questions [55]. It may be considered a limitation that only ten interviews were conducted. However, we argue that a high informational power was reached. Still more interviews might have shown new variations and diversity in womens health literacy experiences being a broad and complex area. Thus more studies are needed to cover the whole area.

Study participants were recruited via an invitation posted on the Aarhus University Hospital Midwifery Clinic’s Facebook page. This introduces the possibility of self-selection bias since the women who responded to the Facebook invitation may have had a higher level of health literacy, interest and motivation than those who did not [56, 57]. This may entail, that our results may be overestimating the womens health literacy. As participants were recruited from a limited geographical area, they may also be unrepresentative of the experiences of women attending antenatal care elsewhere.

The majority of informants were employed in the healthcare system themselves, which may have influenced the results due to better understanding of the organization of the healthcare and terminology. However, the results show that even health professionals experience health literacy struggles when they interact with the healthcare system during pregnancy, which also illustrates the complexity of the context.

## Conclusion

There is a gap between participants’ receipt of professional healthcare information and their ability to translate it into action, along with a concomitant lack of behaviour-change support. Navigation of digital health services also presents a challenge, potentially explained by inadequate e-health literacy. Although apps and digitalisation support successful navigation, they depend on sufficient skills to access and utilise the associated technology. Conflicting health information from social networks versus health professionals also presents a dilemma for pregnant women. Inadequate skills for critically appraising contradictory information, advice and opinions leads to uncertainty, anxiety and disorientation.

There is a need to support pregnant women’s health-promoting behaviour change, navigation of digital services and apps and reconciliation of conflicting health information. This study underlines the need for increased awareness of pregnant women’s health literacy levels and their relationship to antenatal needs. Evidence suggests that a midwifery-led continuity of care may address some of the challenges described by the women in this study. It would be beneficial to explore health professionals’ understanding of health literacy levels among pregnant women.

## Supplementary Information

**Additional file 1: Supplementary Table 1.** Interview guide and according HLQ scales.

## Data Availability

The dataset supporting this study’s conclusions is included within the article.

## Abbreviations

BMI: Body Mass Index
ID: Interpretive Description
WHO: World Health Organization
AUH: Aarhus University Hospital
GDM: Gestational Diabetes Mellitus
