# Supplementary material for: Health literacy and related behaviour among pregnant women with obesity: a qualitative interpretive description study
Source: BMC Pregnancy Childbirth. 2022 Sep 19;22:712. doi: 10.1186/s12884-022-05023-0 (PMC9483451; doi:10.1186/s12884-022-05023-0)
Supplement: Supplementary file 1 — Additional file 1: Supplementary Table 1. Interview guide and according HLQ scales. [file 12884_2022_5023_MOESM1_ESM.docx]

**Supplementary Table 1:** Interview guide and according HLQ scales

| **HLQ scales** | **Interview guide questions** |
| --- | --- |
| *Health information – the ability to actively engage and handle own health, and the ability to act and behave based on health information (a critical approach)*  Scale 2: Having sufficient information to manage my health  Scale 5: Appraisal of health information  Scale 8: Ability to find good health information  Scale 3: Actively managing my health  Scale 9: Understand health information well enough to know what to do  *Health professionals and the health care system*  Scale 1: Feel understood and supported by health professionals  Scale 6: Ability to actively engage with health professionals  Scale 7: Navigating the health care system  *Social support and network*  Scale 4: Social support for health  *Cases*  Breastfeeding  Physical activity  Diet  Birth | Where do you primarily receive information about pregnancy?  (Social network, friends, family, health professionals, social media etc.)   - Other?   Which information have you received about “a good pregnancy”?   - How do you find understandable information about health? - Have you received any specific advice/recommendations from your health professionals?   If yes – which? Can you mention an example of a situation, where you received advice/recommendation?  Which information do you consider important to receive when pregnant in order to have a “good” pregnancy?  When you receive or find information about pregnancy, how do you relate to it?   - How do you decide whether it is something, you will comply/believe/trust? - Have you received information/advice/instruction/guidance where you did not agree with health professionals? - How do you decide whether you believe information, you find, to be true or false? What is essential? - How do you adjust information/advice/recommendations you receive to your everyday life? - If you are in doubt about what health professionals/friends/family mean or says – what do you do? - On the other hand – if you agree with them, what do you do? How do you incorporate or adjust it to your everyday life?   How do you experience the language in the different settings where you receive health information?   - The language from health professionals? - The language in written material? - The language on social media (blogs etc.)? - The language in books?   Whom do you speak with about your pregnancy besides health professionals?   - What did you speak about last time you spoke about your pregnancy? - Do you speak about health information? Things you can and cannot do when pregnant? Can you give some examples?   Who influence your health and your choices about health?   - Has it changed after you became pregnant? - What is health to you?   Do you speak with health professionals about information you receive from other places/persons?   - If yes – how do you include them?   How do you feel about the amount of information you have receive about health in your pregnancy?   - Do you feel like you received/receive sufficient information? - Do you feel like you lacked/lack any information in your pregnancy? - Do you feel sufficient informed to handle your own health?   What do you consider difficult, when you need to find information about your pregnancy?   - Are there any topics you find hard to speak about? - If yes – which? - Is it because you are in doubt about where to find information or because your source of information is not always accessible?   Do you focus on your own health during prengnacy?   - Which activities do you do to stay health during pregnancy? - Do you do anything differently during pregnancy? Have you changed any habbits?   Do you set goals to be healthy during pregnancy?   - If yes – how do you set goals for your pregnancy? - Is it different now that you are pregnant?   Are there barriers, which makes it difficult for you to be healthy during pregnancy?  If you needed to tell me what the health professionals want you to do during pregnancy, what would you say?   - Is it clear to you or not?   Which health professionals have you had contact with in your pregnancy course so far? (Obstetrician, midwife, nurse)  Tell me about last time you met with a health professional in your pregnancy. How did you experience it? What did you talk about?  How do you experience your relation to health professionals?   - Do you feel they are aware of your needs?   Do you have any reflections before appointments with health professional?   - Do you prepare? - If yes, how? - Is it different depending on which health professional you talk to?   How do you experience the dialogue between yourself and health professional?   - Do you feel understood? - What meaning do your relation to a health professional have for the dialogue between you?   How do you experience the information they deliver to you?   - Is it hard to understand? - Do you trust that their advice will benefit you? - Can you give an example of something they said where you felt distrustful?   What do you do if you do not understand health professionals?   - Do you ask if they will explain it again? - How do you feel if you do not understand what you are told?   What is important in order for you to feel understood and supported by health professionals?   - Can you give an example of a situation where a health professional understood and supported you? - Can you give an example of the opposite?   When and how do you have contact with health professionals in the weekdays?  Do you think about which health professionals to contact if you need someone to talk to about your pregnancy? If yes – which thoughts do you have?   - How do you reach them? - Is it clear to you whether it is a midwife or a doctor, who should help you?   What is your experience with navigation in the health care system?   - Both physically, but also in relation to advice, recommendations and the information you receive. - Do you find it hard or easy? - What do you do to navigate and orientate?   How do you know which help or treatments you are entitled to in your pregnancy?   - How do you decide whether to use it?   How do you experience the involvement of your surroundings in your pregnancy? (friends, family, colleagues)  Who support you during pregnancy?   - Can you give an example of how they support you? - Has anyone accompanied you to see the midwife? - Whom do you reach out to, if you feel bad or find something hard?   Whom in your social network do you reach out to if you need help to understand health information during pregnancy? Whom do you reach out to if it concerns your health?  How do you experience the support of your family, friends, colleagues e.g.?  Do you have minimum one person in your social network you feel you can reach out to if you need help?  Which considerations have you had about breastfeeding?   - Do you which to breastfeed? Why/why not? - Where did you find information about breastfeeding? - How have you talked to health professionals/your midwife about breastfeeding? - Whom in your social network have you talked to about breastfeeding? - Can you give a description these your talk?   Which considerations have you had about physical activity during pregnancy?   - Are you physical active during pregnancy? - Has something changed? - Have you searched for information about physical activity in pregnancy? Is yes – where? - Have you talked to health professionals, family or friends about physical activity in pregnancy? If yes – what did you talk about? - Do you experience any barriers or worries about physical activity in pregnancy?   Which consideration do you have about diet during pregnancy?   - Do you have a special focus on diet? Why/why not? - Have you talked to health professionals/midwife about diet? If yes – about what in particular? - Have you searched for information or recommendations about diet during pregnancy? If yes – where?   Which considerations have you had about giving birth?   - How would you like to give birth? - Whom have you talked to about birth? - Do you talk to health professionals about birth? - Which information about birth have you received? Where do you find it/receive it? |
